# Genetic Diversity and Linkage Disequilibrium in Chinese Bread Wheat (*Triticum aestivum* L.) Revealed by SSR Markers

**DOI:** 10.1371/journal.pone.0017279

**Published:** 2011-02-18

**Authors:** Chenyang Hao, Lanfen Wang, Hongmei Ge, Yuchen Dong, Xueyong Zhang

**Affiliations:** Key Laboratory of Crop Germplasm Resources and Utilization, Ministry of Agriculture, The National Key Facility for Crop Gene Resources and Genetic Improvement, Institute of Crop Science, Chinese Academy of Agricultural Sciences, Beijing, China; University of Umeå, Sweden

## Abstract

Two hundred and fifty bread wheat lines, mainly Chinese mini core accessions, were assayed for polymorphism and linkage disequilibrium (LD) based on 512 whole-genome microsatellite loci representing a mean marker density of 5.1 cM. A total of 6,724 alleles ranging from 1 to 49 per locus were identified in all collections. The mean PIC value was 0.650, ranging from 0 to 0.965. Population structure and principal coordinate analysis revealed that landraces and modern varieties were two relatively independent genetic sub-groups. Landraces had a higher allelic diversity than modern varieties with respect to both genomes and chromosomes in terms of total number of alleles and allelic richness. 3,833 (57.0%) and 2,788 (41.5%) rare alleles with frequencies of <5% were found in the landrace and modern variety gene pools, respectively, indicating greater numbers of rare variants, or likely new alleles, in landraces. Analysis of molecular variance (AMOVA) showed that A genome had the largest genetic differentiation and D genome the lowest. In contrast to genetic diversity, modern varieties displayed a wider average LD decay across the whole genome for locus pairs with r^2^>0.05 (*P<0.001*) than the landraces. Mean LD decay distance for the landraces at the whole genome level was <5 cM, while a higher LD decay distance of 5–10 cM in modern varieties. LD decay distances were also somewhat different for each of the 21 chromosomes, being higher for most of the chromosomes in modern varieties (<5∼25 cM) compared to landraces (<5∼15 cM), presumably indicating the influences of domestication and breeding. This study facilitates predicting the marker density required to effectively associate genotypes with traits in Chinese wheat genetic resources.

## Introduction

Bread wheat (*Triticum aestivum* L.) is one of the most important cereal crops worldwide, including China. Wheat is grown in 30 of China's 31 provinces in 10 major agro-ecological zones based on wheat type, growing season, and varietal response to temperature and photoperiod [1], [2]. China is also regarded as one of the centers of diversity of common wheat [3]. Due to a long cultivation history and artificial selection in different ecological regions, about 23,135 domesticated accessions (11,694 landraces and 11,441 modern varieties) constitute the Chinese basic collection conserved in the national genebank [http://icgr.caas.net.cn/cgris_english.html]. Recently, a candidate wheat core collection (5,029 accessions) was established based on geographical regions, ecotypes, and 21 agronomic and botanic characters of the basic collections [3]. According to the utility of core collections in crop wild relatives [4], using a strategy for unlocking genetic potential in crops proposed by Tanksley and McCouch [5], both a core collection, with 1,160 accessions (5% of the national collection) representing 91.5% of the genetic diversity, and a mini core collection consisting of 262 accessions with an estimated 70% representation of the genetic variation in the full collection, were constructed based on 4×10^5^ SSR data-points [6]. This mini core collection is a suitable platform for in-depth evaluation, effective utilization and genetic research in Chinese wheat genetic resources [7], [8].

Linkage disequilibrium (LD), or nonrandom association of alleles between loci (linked or unlinked), is becoming increasingly important for identifying genetic regions associated with agronomic traits [9]–[12]. Recent, genome-wide LD studies were performed on various crop plants, such as maize (*Zea mays* L.) [13]–[15], rice (*Oryza sativa* L.) [16], [17], barley (*Hordeum vulgare* L.) [18], [19], sorghum (*Sorghum bicolor* L. Moench) [20], durum wheat (*T. turgidum* L. var. *durum*) [21] and soybean (*Glycine max* L. Merr.) [22], [23]. From an analysis of 242 genomic SSRs among 43 elite US wheat cultivars, Chao et al., [24] reported genome-wide LD estimates of generally less than 1 cM for genetically linked locus pairs, and that most of the LD was between loci less than 10 cM apart. Somers et al., [25] genotyped 189 bread wheat accessions at 370 loci and 93 durum wheat accessions at 245 loci to examine linkage disequilibrium across the genome, and found that LD mapping of wheat can be performed with simple sequence repeats to a resolution of <5 cM. Most of the diversity and LD analyses on wheat were undertaken at the whole genome level, with the exception of two recent studies at the chromosome level [26], [27]. Breseghello and Sorrells [26] found consistent LD of less than 1 cM for chromosome 2D and about 5 cM in the centromeric region of 5A using 33 and 20 SSR markers, respectively. Horvath et al., [27] suggested that chromosome 3B had a lower diversity than average for the entire B-genome; LD was weak in all materials studied, and marker pairs in significant LD were generally concentrated around the centromere in both arms and at distal positions on the short arm. However, all LD studies to date were based on limited numbers of loci and small sample sizes. It would be valuable to estimate LD decay in bread wheat at the whole genome level and with a larger genetic representation of wheat genotypes.

Based on genotyping of 250 accessions mostly from the Chinese bread wheat mini core collection (70% genetic diversity of the initial collection) using 512 microsatellite loci distributed over all 21 chromosomes, the objectives of this study were: 1) to evaluate the allelic diversity within the Chinese wheat collection; 2) to analyze the population structure and compare the diversity level between landraces and modern varieties; 3) to investigate genetic differentiation of wheat genomes within the two gene pools; and 4) to examine the extent and genomic structure of LD between pairs of SSR markers on both genome-wide and chromosome scales. The results of this study should describe the level of genetic diversity and linkage disequilibrium decay of a representative Chinese collection for breeding and genetic research, and provide a molecular basis to enrich genetic diversity of bread wheat worldwide.

## Results

### Overall Diversity of Chinese Wheat Collections

The genetic characteristics of the 250 member Chinese wheat mini core collection based on 512 microsatellite loci are listed in Table 1. Among 512 SSR loci, 99.4% (509) were polymorphic with just 3 being monomorphic. A total of 6,724 alleles ranging from 1 to 49/locus were detected. PIC values ranged from 0 to 0.967 and the total number of rare alleles with a frequency of less than 5% reached 4,424 (65.8%), indicating that many new alleles occurred in the mini core collection. As expected from the way in which the collection was constructed, the combination of mean genetic richness (13.1) and genetic diversity index (0.650) indicated high levels of polymorphism.

**Table 1:** Allelic diversity of Chinese wheat collections at 512 whole-genome SSR loci.

Parameter	Total (mean)
No. of accessions	250
No. of loci	512
No. of polymorphic loci	509
Allele range	1–49
*PIC* range	0–0.967
No. of alleles	6724
No. of rare alleles (<5%)	4424 (65.8%)
Mean genetic richness	13.1±0.340
Genetic diversity index	0.650±0.010

### Genetic Structure of the Wheat Collection

**Figure 1:**
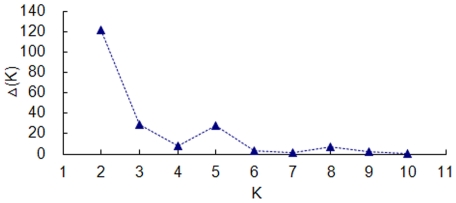
Estimation of the number of populations for K ranging from 1 to 11 by calculating delta K values.

Population structure of whole collection was investigated using a Bayesian clustering approach, to infer the number of clusters (populations) with STRUCTURE v2.2 software [28]. The structure result at K = 2 was the best separator providing the highest delta k value (Figure 1).

**Figure 2:**
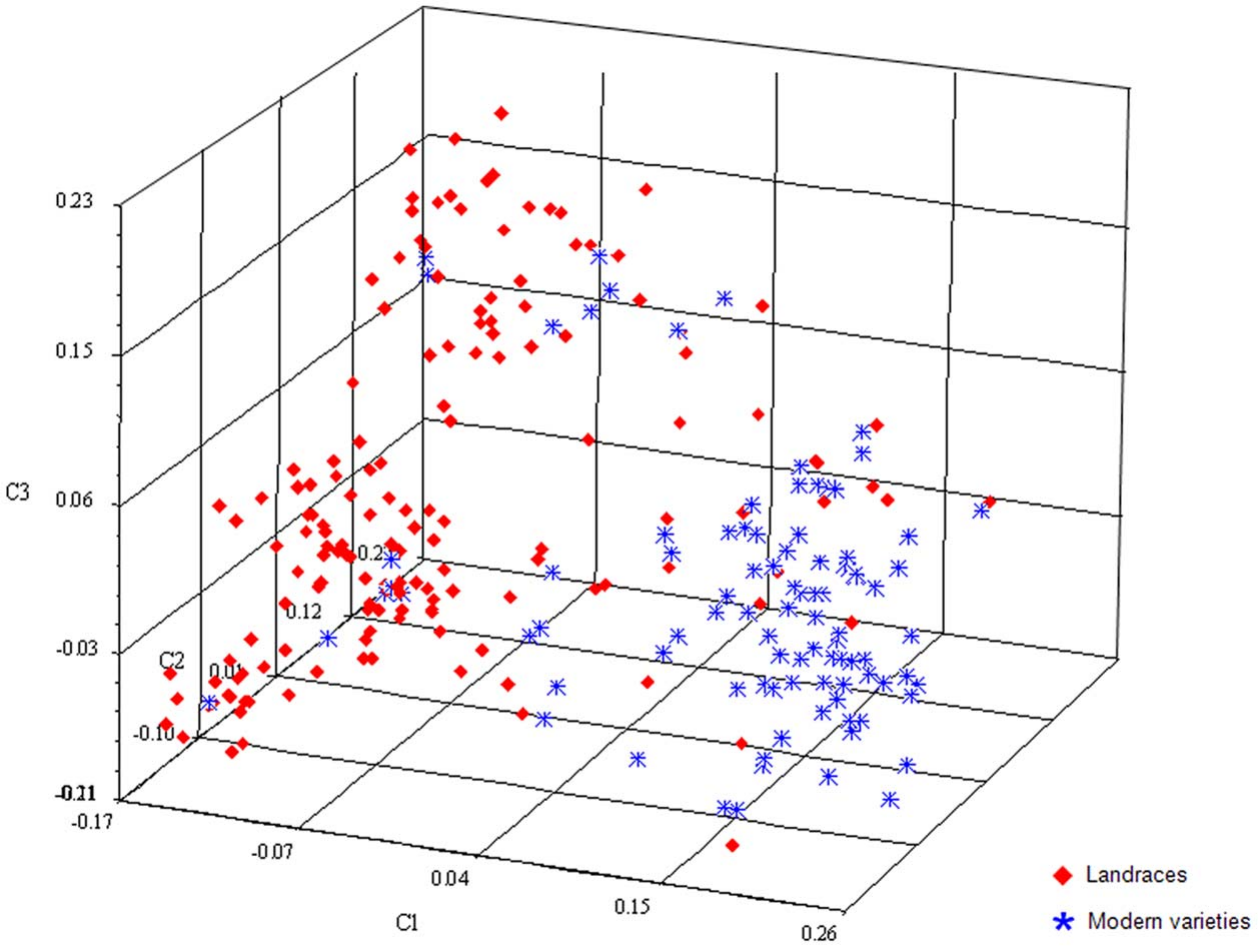
Principal coordinate analysis of 250 Chinese wheat accessions based on 512 microsatellite markers indicating separation of the landrace (red) and modern variety (blue) sub-groups.

Principal coordinate analysis also indicated two major sup-groups within the mini core collection (Figure 2). A large proportion of accessions formed one sub-group indicated to the left, and the other sub-group (right) included accessions predominantly in the modern cultivar sub-group. The greater scattering of the landrace sub-group indicated its higher diversity. Overlapping between the two gene pools indicated by intermediates in both sub-groups, was probably caused by breeding activities in the 1940s–1950s. During this period new varieties were produced from landrace×introduced cultivar hybrids [2]. Consistent with previous studies [29]–[31], principal coordinate analysis of the mini core collection clearly indicated that Chinese landraces and modern varieties comprised separate sub-groups of genotypes.

### Genetic Characteristics within the Landrace and Modern Variety Sub-groups

**Figure 3:**
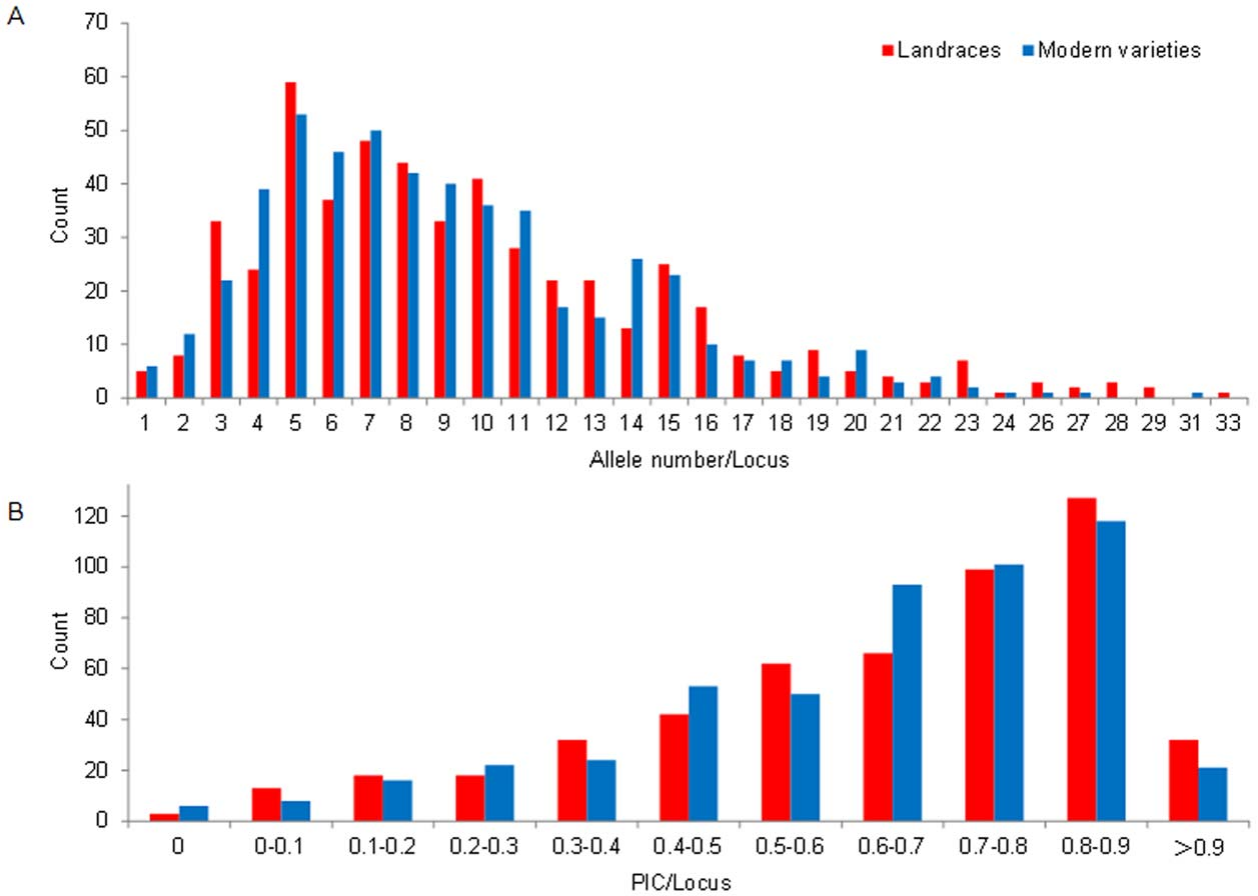
Distribution of allele numbers per locus (A) and *PIC* per locus (B) calculated using rarefacted samples of 68 accessions per sub-group for all SSR loci.

The basic statistics of genetic diversity between the landrace and modern variety sub-groups at the genome level are listed in Table 2. In total, 6,122 alleles ranging from 1 to 46 were identified at 512 SSR loci in the landrace sub-group, compared with 5,004 alleles ranging from 1 to 35 in the modern variety sub-group. Similarly, private alleles of the landrace were 1,720 (28.1%), but just 602 (12.0%) for modern varieties. Correspondingly, there were 3,833 (62.6%) rare alleles with frequencies <5% for the landrace sub-group whereas this number was 2,788 (55.7%) for modern varieties, indicating higher genetic variation in novel alleles for the landraces than in modern varieties. Allele number per locus (Figure 3A) and *PIC* per locus (Figure 3B) for all SSR loci were continuously distributed in both landrace and modern varieties. Within the sub-groups allelic numbers per locus ranged from 4 to 13, and *PIC* values ranged from 0.6 to 0.9, indicating high polymorphism levels in both sub-groups. Both mean genetic richness (12.0) and genetic diversity indices (0.640) of the landraces were higher than those for modern varieties at 9.8 and 0.628, respectively. Consistent with landraces having higher diversity than modern varieties at the whole genome level, these relationships were retained when the three genomes were individually compared. To eliminate the influence of sample size on evaluations of genetic diversity, allelic richness calculated following rarefaction on samples of 68 accessions per sub-group likewise indicated that landraces had higher genetic diversity than modern varieties (Table 2).

**Table 2:** Comparison of genetic diversity between the landrace and modern variety sub-groups at the genome level.

Parameter	A		B		D		Whole genome	
	Mv	L	Mv	L	Mv	L	Mv	L
No. of loci	149	149	177	177	186	186	512	512
Allelic range	1–35	2–46	1–24	1–38	1–28	1–35	1–35	1–46
No. of alleles	1638	2010	1778	2180	1588	1932	5004	6122
No. of rare alleles (<5%)	963	1330	993	1366	832	1137	2788	3833
Private alleles	212	584	222	624	168	512	602	1720
Mean genetic richness	11.0±0.503	13.5±0.658	10.0±0.364	12.3±0.508	8.5±0.351	10.4±0.456	9.8±0.235	12.0±0.313
Genetic diversity indices	0.661±0.018	0.674±0.018	0.644±0.017	0.650±0.018	0.587±0.016	0.604±0.017	0.628±0.010	0.640±0.010
Allelic richness^a^	10.4	11.7	9.7	10.8	8.3	9.3	9.5	10.7

Mv: modern variety, N = 93; L: landrace, N = 157.

a Calculated using rarefacted samples of 68 accessions per sub-group.

A comparative analysis of genetic characteristics between sub-groups was performed at the chromosome level (Table 3). For all chromosomes, the total number of alleles for landraces ranging from 130 to 450 was higher in modern varieties (99 to 361). Again, comparing 68 accessions from each sub-group confirmed that landraces had more alleles per locus than modern varieties at the chromosome level. Except for chromosomes 1B, 5A and 5B, landraces had much higher *PIC* values than modern varieties for all other chromosomes. The total number of private alleles for landraces ranging from 26 to 140 was higher than that of modern varieties (6 to 49) on all individual chromosomes, as well as the distribution of rare alleles in the two gene pools. The mean *F_st_* value for all chromosomes was 0.021 ranging from 0.010 to 0.035 between landraces and modern varieties. Chromosome 3A (0.035) provided the highest genetic differentiation and 1D (0.010) the lowest (Table 3).

**Table 3:** Total numbers of alleles, mean *PIC* values, private alleles and *F_st_* within and between landraces and modern varieties at the chromosome level.

Chr.	No. of loci	No. of alleles		Allelic richness^a^		*PIC*		Rare alleles (<5%)		Private alleles		*F_st_*
		Mv	L	Mv	L	Mv	L	Mv	L	Mv	L	
1A	20	189	235	8.7	9.6	0.590	0.614	110	155	24	70	0.029
1B	24	251	303	8.9	9.4	0.649	0.629	147	201	29	81	0.020
1D	23	202	230	8.1	8.5	0.580	0.598	107	134	32	60	0.010
2A	22	243	297	9.8	10.7	0.690	0.733	145	187	32	86	0.025
2B	28	236	268	7.7	8.0	0.579	0.590	122	156	36	68	0.019
2D	31	358	442	10.7	12.2	0.653	0.658	209	293	32	116	0.012
3A	21	250	295	10.7	11.3	0.719	0.745	150	185	31	76	0.035
3B	33	358	449	10.4	11.6	0.656	0.662	213	299	49	140	0.022
3D	24	220	237	8.7	8.6	0.603	0.622	123	132	28	45	0.017
4A	15	166	207	9.0	10.0	0.592	0.604	103	147	25	66	0.019
4B	18	167	189	7.9	8.5	0.592	0.607	97	115	22	44	0.015
4D	15	99	140	5.2	6.5	0.530	0.552	42	81	6	47	0.011
5A	30	325	410	9.8	11.0	0.659	0.630	186	287	40	125	0.027
5B	30	280	378	8.9	10.9	0.634	0.616	140	243	32	130	0.022
5D	47	361	450	7.3	8.4	0.564	0.583	183	267	40	129	0.013
6A	15	172	219	9.8	11.3	0.704	0.732	98	141	15	62	0.016
6B	16	168	213	8.7	10.5	0.700	0.733	92	119	11	56	0.028
6D	18	114	130	5.8	6.1	0.547	0.550	50	61	10	26	0.022
7A	26	293	347	9.5	10.3	0.662	0.672	171	228	45	99	0.025
7B	28	318	380	9.9	10.8	0.704	0.731	182	233	43	105	0.023
7D	28	234	303	7.7	9.2	0.599	0.630	118	169	20	89	0.032
Range	15–47	99–361	130–450	5.2–10.7	6.1–12.2	0.530–0.719	0.550–0.745	42–213	61–299	6–49	26–140	0.010–0.035
Mean	24.4	238.3	291.5	8.7	9.7	0.629	0.642	132.8	182.5	28.7	81.9	0.021

Mv: modern variety, N = 93; L: landrace, N = 157.

a Calculated using rarefacted samples of 68 accessions per sub-group.

**Figure 4:**
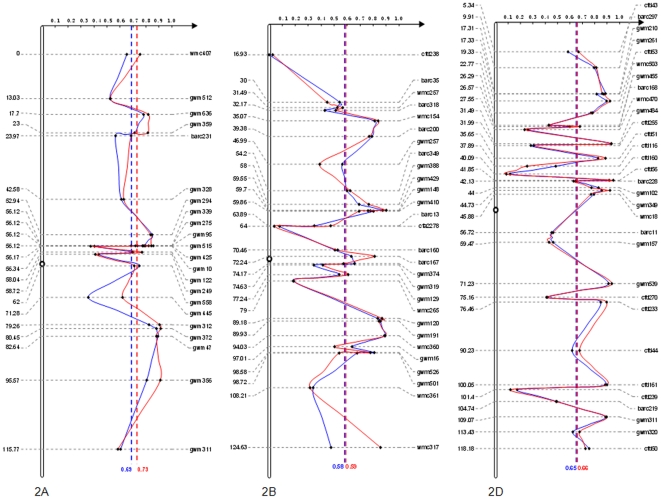
PIC distribution of SSR loci between landrace and modern variety sub-groups on chromosomes 2A, 2B and 2D. Blue curve shows PIC changes in modern varieties, and red curve shows changes in the landrace sub-group. The blue broken line represents average *PIC* value for all SSR loci in modern varieties, and the red broken line is for the landraces. The detailed mean *PIC* values are given at the bottom of each broken line. Genetic positions (cM) of SSR loci are shown on the left of each chromosome.

By comparing genetic diversities with the parameter *PIC* value between the landrace and modern variety sub-groups on homologous group 2 chromosomes (Figure 4), we found that genetic differentiation between them might not be on a genome-wide scale, but rather on selected loci or chromosome intervals, exemplified by the chromosome 2A interval *gwm558*-*gwm312*. Within the region, the locus shows a large reduction in diversity with selection as a one of several possible explanations in modern varieties shown by the lower *PIC* value. Similar comparisons between all chromosomes can be deduced from Figure S2.

Comparisons of the landrace and modern variety sub-groups on the basis of genomes are shown in Table 4. Evaluated by Shannon's information index (*I*) and genetic distance (*GD*), the A genome was the most diverse and the D genome was the least. Gene flow estimated from *F_st_* (*N_m_*) and genetic identity (*GI*) placed the A genome lowest among the three genomes, while the D genome ranked first. This indicated that the largest genetic differentiation between the two sub-sets was within the A genome with the least differences in the D.

**Table 4:** Genetic differences between landraces and modern varieties in different genomes.

Genome	I	GD	F_st_	N_m_	GI
A	1.769	0.120	0.025	9.590	0.887
B	1.683	0.093	0.022	11.161	0.912
D	1.496	0.056	0.017	14.457	0.946
whole	1.650	0.090	0.021	11.736	0.915

I: Shannon's information index.

GD: genetic distance.

N_m_: gene flow estimated from N_m_ = 0.25 (1-F_st_)/F_st_.

GI: genetic identity.

Analysis of molecular variance (AMOVA) between the landraces and modern varieties by genomes was also carried out (Table 5). All sources of variation were highly significant (*P<0.001*) and more than 95% of the variance was explained by differences within the A, B and D genomes, whereas only a small part of the overall variance (less than 5%) could be attributed to differences between landraces and modern varieties. The AMOVA analysis also revealed similar structures of genetic differentiation consistent with the basic statistics (Table 3) when comparing the three genomes. The amount of variation in the A genome (4.73%) was higher than that of the B (4.25%) and D (3.05%) genomes again indicating that the A genome had the largest molecular variance and D genome the lowest between the two sub-groups.

**Table 5:** Analysis of molecular variance (AMOVA) between landraces and modern varieties within genome.

Genome	Source of variation	*df*	Variance component	% variation
A	Among sub-groups	1	2.09*** Va	4.73
	Within sub-groups	498	42.07*** Vb	95.27
	Total	499	44.16	
B	Among sub-groups	1	1.98*** Va	4.25
	Within sub-groups	498	44.52*** Vb	95.75
	Total	499	46.50	
D	Among sub-groups	1	1.50*** Va	3.05
	Within sub-groups	498	47.74*** Vb	96.95
	Total	499	49.24	
Whole	Among sub-groups	1	5.57*** Va	3.98
	Within sub-groups	498	134.34*** Vb	96.02
	Total	499	139.91	

***significant at *P<0.001.*

### Linkage Disequilibrium at the Whole Genome Level

**Figure 5:**
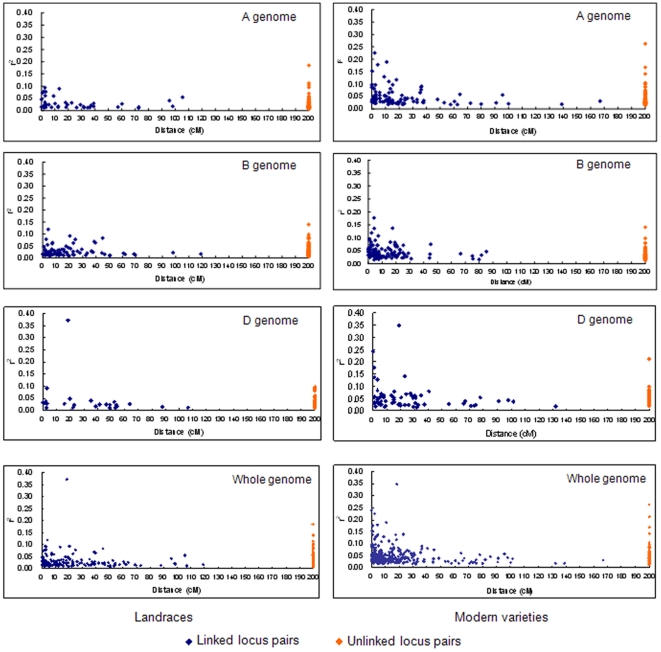
Plots of significant r^2^ values (*P<0.001*) between locus pairs on A, B, D and whole genomes in landraces and modern varieties.

After deletion of some low frequency alleles (<5%) in both sub-groups, 495 loci were chosen to evaluate the extent of linkage disequilibrium (LD) on a whole genome level in the two wheat gene pools (Table 6). There were 143 (149), 171 (177) and 181 (186) loci on each of the A, B and D genomes available for LD evaluations. Across all 495 loci, 6,171 possible linked locus pairs (in the same linkage groups) and 116,094 unlinked locus pairs (from different linkage groups) could be detected in both sub-groups. Among linked locus pairs, 149 (2.41%) of 4,577 compared were in LD at the *P<0.001* level for landraces, whereas there were 275 (4.46%) of 4,736 in significant LD among modern varieties. In addition, the numbers of locus pairs in LD with r^2^>0.1 or r^2^>0.2 in modern varieties were also relatively higher than those in landraces. Furthermore, the mean r^2^ for all significant LD (*P<0.001*) in modern varieties (0.049, ranging from 0.015 to 0.348) was still larger than for landraces (0.030, ranging from 0.008 to 0.371). Although the landraces possessed more significant unlinked locus pairs (*P<0.001*) than modern varieties, i.e. 1,509 (1.30%) vs 1,019 (0.88%), modern varieties had higher r^2^ value in other parameters. LD comparisons on a genome basis showed similar trends of higher LD in modern varieties than in landraces, even though there were very low genome-wide LD levels in both sub-groups. Plots of significant r^2^ values (*P<0.001*) between locus pairs in different genomes of the two sub-groups (Figure 5) further supported earlier results.

**Table 6:** SSR locus pairs in significant (*P<0.001*)linkage disequilibrium (LD) and r^2^ values between Chinese landraces and modern varieties.

	No. of locus pairs in LD											The extent of LD	
Genome/Sample	No. of loci	Linked locus pairs					Unlinked locus pairs					Linked locus pairs	Unlinked locus pairs
		Possible	Observed	Significant/%	r^2^>0.1/%	r^2^>0.2/%	Possible	Observed	Significant/%	r^2^>0.1/%	r^2^>0.2/%	Mean r^2^/Range	Mean r^2^/Range
A													
Landrace	143	1489	1011	43 (2.89)	0 (0)	0 (0)	8664	5871	182 (2.10)	1 (0.01)	0 (0)	0.030 (0.010–0.094)	0.021 (0.008–0.185)
Modern variety			1086	102 (6.85)	4 (0.27)	1 (0.07)		6260	130 (1.50)	4 (0.05)	1 (0.01)	0.049 (0.017–0.225)	0.037 (0.015–0.262)
B													
Landrace	171	2110	1543	81 (3.84)	1 (0.05)	0 (0)	12425	9077	207 (1.67)	1 (0.01)	0 (0)	0.028 (0.008–0.119)	0.023 (0.005–0.140)
Modern variety			1519	112 (5.31)	3 (0.14)	0 (0)		9107	95 (0.76)	0 (0)	0 (0)	0.046 (0.015–0.177)	0.032 (0.012–0.098)
D													
Landrace	181	2572	2023	24 (0.93)	1 (0.04)	1 (0.04)	13718	10893	81 (0.59)	0 (0)	0 (0)	0.040 (0.009–0.371)	0.022 (0.008–0.094)
Modern variety			2131	65 (2.53)	5 (0.19)	2 (0.08)		11352	73 (0.53)	1 (0.01)	1 (0.01)	0.054 (0.013–0.348)	0.041 (0.017–0.212)
Whole gnome													
Landrace	495	6171	4577	149 (2.41)	2 (0.03)	1 (0.02)	116094	85681	1509 (1.30)	7 (0.01)	1 (0.00)	0.030 (0.008–0.371)	0.022 (0.005–0.279)
Modern variety			4736	275 (4.46)	13 (0.21)	3 (0.05)		88646	1019 (0.88)	10 (0.01)	3 (0.00)	0.049 (0.015–0.348)	0.035 (0.012–0.348)

a Loci on the same linkage group.

b Loci from different linkage groups.

**Figure 6:**
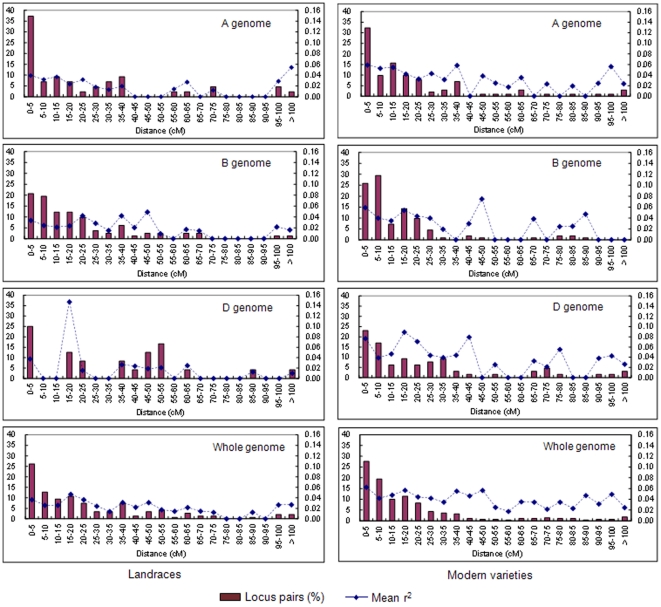
Percentage of locus pairs in significant (*P<0.001*) LD and mean r^2^ among distance intervals for A, B, D and whole genomes in the landrace and modern variety sub-groups.

To reveal LD decay distances in the two sub-groups on a whole genome scale, we plotted percentage of locus pairs with significant (*P<0.001*) LD and mean r^2^ among distance intervals for each gene pool (Figure 6). The percentage of locus pairs in significant (*P<0.001*) LD decreased as genetic distance increased, and there were higher scales of significant LD within 10 cM generally. However, mean r^2^ along distance intervals presented an uneven distribution, i.e. there were some points with relatively higher mean r^2^ at larger intervals. Considering lower LD values for our samples (Table 6, Figure 6), we determined average LD decay distance in the different genomes for locus pairs with r^2^>0.05 at *P<0.001* in the landrace and modern variety sub-groups (Table 7). Mean LD decay distance for landraces at a whole genome level was <5 cM, with higher LD decay distances in modern varieties for the same genomes. For B, D and the whole genomes, the decay distances were increased to 5–10 cM, but 15–20 cM for the A genome in the modern variety sub-group, which might be caused by demographic history for genome-level changes on modern varieties.

**Table 7 pone-0017279-t007:** Average LD decay distance in different genomes for locus pairs with r^2^>0.05 at *P<0.001* in landraces and modern varieties.

Genome	Landraces	Modern varieties
A	<5	15–20
B	<5	5–10
D	<5	5–10
Whole	<5	5–10

### Linkage Disequilibrium at the Chromosome Level

After scanning the extent and structure of LD between landraces and modern varieties on a whole genome scale, the same evaluations were performed at the single chromosome level based on 495 SSR loci in the two gene pools (Tables 8 and 9). Comparing SSR locus pairs in significant (*P<0.001*) LD and mean r^2^ values between landraces and modern varieties (Table 8), the number of mean locus pairs in significant LD was 7.1 (2.41%) ranging from 1 to 30 for the landrace sub-group, and 13.1 (4.47%) ranging from 2 to 35 in the modern variety sub-group. Correspondingly, mean r^2^ of the landrace sub-group was only 0.033 ranging from 0.011 to 0.140, whereas in the modern variety sub-group it was 0.053 ranging from 0.026 to 0.194. At the individual chromosome level, except for chromosomes 1A and 6D, the modern varieties had more SSR locus pairs in significant LD. Nevertheless, the mean r^2^ for modern varieties was still larger than for landraces for all chromosomes except 4A and 4D. Therefore, compared with the landrace sub-group, the modern variety gene pool still had higher numbers of SSR locus pairs in significant LD and higher mean r^2^ values for almost all wheat chromosomes. However, these parameters were not compared among all chromosomes within the same gene pool because of a big difference of loci selected on each chromosome in the present study.

**Table 8 pone-0017279-t008:** SSR locus pairs in significant (*P<0.001*) LD on all 21 chromosomes and r^2^ values between Chinese landraces and modern varieties.

Chr.	No. of loci	Possible	Landraces				Modern varieties			
			Observed	Significant (%)	Mean r^2^	r^2^ range	Observed	Significant (%)	Mean r^2^	r^2^ range
1A	19	171	114	11 (6.43)	0.027	0.010–0.072	124	5 (2.92)	0.075	0.024–0.225
1B	24	276	196	3 (1.09)	0.014	0.009–0.021	189	10 (3.62)	0.032	0.015–0.060
1D	22	231	184	1 (0.43)	0.012	0.012	190	7 (3.03)	0.038	0.026–0.054
2A	22	231	154	12 (5.19)	0.032	0.011–0.094	158	23 (9.96)	0.044	0.021–0.110
2B	26	325	269	14 (4.31)	0.048	0.011–0.119	238	15 (4.62)	0.069	0.023–0.177
2D	30	435	274	2 (0.46)	0.024	0.014–0.034	280	5 (1.15)	0.026	0.015–0.055
3A	21	210	160	7 (3.33)	0.014	0.009–0.018	169	14 (6.67)	0.046	0.017–0.096
3B	32	496	332	10 (2.02)	0.015	0.009–0.026	378	14 (2.82)	0.035	0.017–0.072
3D	24	276	231	1 (0.36)	0.011	0.011	236	4 (1.45)	0.043	0.024–0.066
4A	12	66	41	3 (4.55)	0.044	0.014–0.089	52	3 (4.55)	0.034	0.024–0.042
4B	17	136	96	2 (1.47)	0.025	0.020–0.031	93	5 (3.68)	0.033	0.024–0.045
4D	14	91	75	1 (1.10)	0.090	0.090	87	4 (4.40)	0.049	0.020–0.129
5A	29	406	255	6 (1.48)	0.046	0.016–0.077	303	31 (7.64)	0.060	0.017–0.189
5B	29	406	325	30 (7.39)	0.027	0.008–0.079	304	35 (8.62)	0.047	0.023–0.137
5D	46	1035	846	12 (1.16)	0.026	0.009–0.046	904	28 (2.71)	0.056	0.016–0.241
6A	15	105	75	1 (0.95)	0.017	0.017	79	12 (11.43)	0.037	0.017–0.065
6B	16	120	85	9 (7.50)	0.016	0.010–0.024	80	13 (10.83)	0.052	0.019–0.136
6D	18	153	133	3 (1.96)	0.140	0.024–0.371	146	2 (1.31)	0.194	0.039–0.348
7A	25	300	214	4 (1.33)	0.026	0.013–0.040	204	15 (5.00)	0.043	0.019–0.152
7B	27	351	240	14 (3.99)	0.029	0.010–0.064	237	20 (5.70)	0.038	0.016–0.064
7D	27	351	280	3 (0.85)	0.013	0.010–0.016	288	11 (3.13)	0.060	0.017–0.176
Mean	23.6	293.9	218.0	7.1 (2.41)	0.033	0.016–0.064	225.7	13.1 (4.47)	0.053	0.021–0.126

**Table 9:** Average LD decay distance in different chromosomes in the landrace and modern variety sub-groups for locus pairs with r^2^>0.05 at *P<0.001*.

Chr.	Landraces	Modern varieties
1A	<5	5–10
1B	<5	<5
1D	<5	<5
2A	<5	5–10
2B	5–10	10–15
2D	<5	<5
3A	<5	<5
3B	<5	<5
3D	<5	<5
4A	<5	<5
4B	<5	<5
4D	<5	5–10
5A	10–15	20–25
5B	<5	5–10
5D	<5	5–10
6A	<5	5–10
6B	<5	<5
6D	<5	<5
7A	<5	5–10
7B	<5	<5
7D	<5	20–25

Average LD decay distances on different chromosomes for locus pairs with r^2^>0.05 at *P<0.001* in the two sub-groups are depicted in Table 9. It was interesting that LD decay distance was <5 cM for 19 of 21 chromosomes in the landrace sub-group, but 5–10 cM for 2B and 10–15 cM for 5A. In the modern variety sub-group, chromosomes 1B, 1D, 2D, 3A, 3B, 3D, 4A, 4B, 6B, 6D, 7B had *<*5 cM LD decay distances similar to the landraces, but the other 10 chromosomes showed wider LD decay distances than those of the landraces, especially the values 20–25 cM for 5A and 7D. These general descriptions of LD decay distance provide important information concerning decisions on marker densities for future association analyses at the chromosome level, and also guidance on different strengths of selective signals in breeding imprinted on each chromosome.

## Discussion

### Genetic Relationship and Population Structure

In our previous studies, 43 cornerstone breeding parents used before 1980 and widely grown varieties in current use in China [29], 96 random samples with maximized genetic diversity [30], a 340 candidate core collection from the Northwestern Spring Wheat Region [31], and a 1,110 member Chinese core collection [6], consistently demonstrated that Chinese landraces and modern varieties are relatively independent genetic sub-groups.

To address possible limitations in the number of loci used in above-mentioned studies, we employed 512 microsatellite loci identifying 6,724 alleles to obtain a genetic structure of Chinese wheat genetic resources using principal coordinate analysis and Bayesian clustering approaches. The larger number of alleles identified in 512 SSR loci also indicated that individual microsatellite loci have higher information content [32]–[34]. Using a relatively large set of molecular data-points, the Chinese mini core collection was divided into two major sub-groups basically, landraces and modern varieties. This was considered consistent with the history of Chinese wheat breeding. Within each sub-group there were some intermediate genotypes. Adopting with a threshold probability >0.50 to fitting one of the clusters [24], [26], 78 of 93 modern varieties were clearly assigned to one sub-group and 135 of 157 landraces to the other. Examples of the 37 varieties with a lower probability (*<*0.50) of fitting either sub-group included Lianglaiyoubaipixiaomai (Inner Mongolia), Bihongsui (Inner Mongolia), Mingxian 169 (Shanxi), Shite 14 (Hebei), Fuzhuang 30 (Shaanxi), and Jingyang 60 (Shaanxi). Even though they were arbitratrily classified into modern varieties, most of them were selections of landraces or were from hybrid progeny of landraces [2], [6], and still retained most of the genetic characteristics of landraces.

### Genetic Diversity in Chinese Wheat Gene Pools

Allelic diversity analysis in this study revealed that the total number of alleles amplified at 512 SSR loci in 250 accessions was up to 6,724 (13.1 alleles per locus on average, ranging from 1 to 49), and polymorphism information content values ranged from 0 to 0.967 (mean 0.650). These values were higher than the previously reported estimates of SSR marker diversity in wheat [24]–[26], [35], [36]. And, allele number was ranged from 4.81 to 10.5 and mean PIC value from 0.46 to 0.62 for above-mentioned studies. On the other hand, a genetic diversity of 0.77 and 18.1 alleles [37], 14.5 alleles and a genetic diversity of 0.662 [38], and, 23.9 alleles per locus over 38 SSR markers [39] were also reported. Comparatively, the high SSR allele diversity found in the minicore collection approximately reflects the genetic representation of the entire set of Chinese wheat collections. It is very interesting that there were a total of 4,424 alleles with frequencies of less than 5% among all accessions, and these so-called rare alleles represented 65.8% of all alleles detected. Similar with common alleles, rare variants or new alleles unselected artificially also played an important role in genome-wide genetic research [40].

The amounts of genetic diversity in the two gene pools and PIC values were significantly different at both the genome (Table 2) and individual chromosome (Table 3) levels, in terms of allelic richness calculated using equivalent numbers of accessions from each sub-group. Results of allelic diversity using 512 SSR markers indicated that the landraces (mean genetic richness: 12.0; genetic diversity index: 0.640; allelic richness: 10.7) actually had higher genetic diversity than modern varieties (mean genetic richness: 9.8; genetic diversity index: 0.628; allelic richness: 9.5). This was consistent with a previous study analyzing 1,160 a Chinese wheat core collection composed of 762 landraces and 348 modern varieties using 78 microsatellite markers [6]. Like the whole genome, similar results were obtained for individual genomes and chromosomes. This implied there were more potentially rare variants or new alleles in the landrace gene pool. Obviously, these could be of value for genetic research or breeding.

China has a more than four millennia history of wheat cultivation, and landraces became isolated because of limited transportation in earlier times [6]. Scientific breeding in China can be traced back only 50–90 years [2]. The history of Chinese wheat breeding shows that new varieties were usually selected from landraces in the early period, later from crosses between landraces and introduced varieties, and more recently from crosses between Chinese modern varieties. In this study, genetic analyses including Shannon's information index (*I*), genetic distance (*GD*), genetic differentiation coefficient (*F_st_*), and analysis of molecular variance (AMOVA) between the landrace and modern variety sub-groups for different genomes suggested that the A genome (4.73%) was significantly more variable than the B (4.25%) and D (3.05%) genomes, indicating stronger selective pressure on the A genome during Chinese wheat breeding. However, a selection sweep imprinted across genomes suggested that some important loci or chromosomal intervals rather than whole genomes (or chromosomes) were responsible for the differences (Figure 4). This is consistent with findings in sunflower reported by Chapman et al., [41].

### LD Level in Chinese Bread Wheat

A number of LD mapping studies in wheat were performed at the genome or chromosome levels [24]–[27], so it is important to examine the extent of LD in Chinese wheat genomes. This determines the genetic distances over which LD will decay back to a random association of alleles and facilitates prediction of marker density needed to effectively associate genotypes with traits [11]. In the present study 512 SSR loci with a mean marker density of 5.1 cM per locus, ranging from 2.2 to 9.4 cM for all 21 chromosomes, were used to measure LD in Chinese wheat genetic resources at both the genome and chromosome levels (Tables 6–9, Figures. 5 and 6).

Population structure is one of several important factors that have strong influences on LD, besides recombination, mutation, population size, genetic drift, population mating pattern, admixture, and selection [10]. The presence of population stratification and an unequal distribution of alleles within groups can result in nonfunctional, spurious associations [42]. In our LD estimations, we took into account the effect of population structure by subdividing the genetic resources into two main gene pools, i.e. Chinese landraces and modern varieties, which were validated by the results of genetic structure with the software STRUCTURE v2.2 [28] (Figure 1) and principal coordinate analysis using NTSYS-pc version 2.1 software [43] (Figure 2).

LD decay distance with r^2^>0.05 at *P<0.001* was consistent for all linked locus pairs in each gene pool. Mean LD decay distance for the landraces at the whole genome level was *<*5 cM, whereas higher values applied in the modern varieties. In detail, as to B, D and whole genomes, the decay distance increased to 5–10 cM, and 15–20 cM for A genome in the modern variety sub-group, possibly due to demographic history for genome-level changes [44]. At a chromosome level, LD decay distance was *<*5 cM for 19 of the 21 chromosomes in the landrace sub-group, but 5–10 cM for 2B and 10–15 cM for chromosome 5A. As for the modern variety sub-group, chromosomes 1B, 1D, 2D, 3A, 3B, 3D, 4A, 4B, 6B, 6D, and 7B had *<*5 cM values similar to the landraces, but the other 10 chromosomes showed wider LD decay distances extending to 20–25 cM for 5A and 7D. This indicated that these two chromosomes may carry more QTLs or genes related to important agronomic traits that were strongly selected in breeding [12]. Our results further demonstrated population-dependent and genome-dependent LD characteristics in comparison with genome-wide LD estimates of less than 1 cM in 43 US wheat elite cultivars [24], *<*5 cM for LD decay distance across the genome among 189 bread wheat accessions from western Canadian wheat breeding programs [25], less than 1 cM on chromosome 2D and about 5 cM in the centromeric region of 5A in 95 soft winter wheat from the eastern United States [26].

In general, a significance level of *P*<*0.001* was adopted as a comparison threshold. Thus, Somers et al., [25] found that bread and durum wheat collections had 47.9% and 14.0% of all locus pairs in significant LD, but within the groups only 0.9% (bread wheat) and 3.2% (durum wheat) of locus pairs were in LD with r^2^>0.2. Malysheva-Otto et al., [19] also showed that 100% of locus pairs in significant LD based on r^2^>0.05 at P<0.001 in a wide set of barley varieties, but this fell to 45% in a European 2-rowed spring barley subgroup. This indicated that the number of loci in significant LD, as well as the extent of LD, was clearly dependent on the population structure and on different genomes. In the present study, 2.41% and 4.46% of locus pairs for the Chinese landrace and modern variety gene pools were in significant LD (*P<0.001*) on a whole genome level, but only 0.02% and 0.03% were in LD based on r^2^>0.2 within the two gene pools (Table 6). The extremely low values of LD in the two clusters can be seen as evidence that many of the recombination events in past breeding history have been maintained and fixed in homozygous self-fertilizing bread wheat, as well as a reflection of the higher genetic diversity that is maintained in the mini core collection in Chinese wheat genetic resources. Understanding the patterns of LD across the genome will facilitate prediction of marker densities required for efficient association of genotypes with traits in Chinese wheat genetic resources at both the genome and chromosome levels.

## Materials and Methods

### Plant Materials

A total of 250 wheat accessions were used in the present study. These included 93 modern varieties and 157 landraces (), among which 245 (98%) were from the Chinese wheat mini core collection constructed in our group [6], [31]. This collection representing just 1% of the national collection has more than 70% of its genetic diversity.

### Microsatellite Analysis

Genomic DNA of all materials was extracted using lyophilized pooled young leaves of ten seedlings following Sharp et al., [45]. A total of 512 pairs of SSR primers with good genome coverage were selected to genotype the collection. The primers comprised 212 GWM [46], 114 BARC [47], 89 WMC [48], 66 CFD, 18 CFA plus 2 GPW [INRA, http://wheat.pw.usda.gov/ggpages/SSRclub/], 10 GDM [49] and 1 PSP primer set. Genetic distance (cM) of each locus on a consensus map obtained from the Komugi wheat genetic resources database [http://www.shigen.nig.ac.jp/wheat/komugi/top/top.jsp] (Table S2, Figure S1). In total, these SSR loci covered 2,631.3 cM with a mean genetic distance of 5.1 cM between adjacent loci (Table S3). More information concerning these wheat microsatellite markers is available in the GrainGenes 2.0 database [http://wheat.usda.gov/GG2/index.shtml]. Fluorescence-labeled primers that were relatively evenly distributed on the 21 wheat chromosomes were synthesized at Applied Biosystems Company. An ABI 3730 Analyzer (Applied Biosystems) was used to capture amplification products by a fluorescence detection system for microsatellite markers. More detailed experimental procedures are given in Hao et al., [31]. Fragment sizes were evaluated using GeneMapper v3.7 software (Applied Biosystems), and the molecular data-points for all SSR markers are listed in Table S4.

### Data Analysis

Population structure analysis for the 250 Chinese wheat accessions was performed using the molecular datasets of 512 whole-genome SSR markers with STRUCTURE v2.2 software [28]. We adopted the “admixture model”, burn-in period equal to 50,000 iterations and a run of 100,000 replications of Markov Chain Monte Carlo (MCMC) after burn in. For each run, 5 independent runs of STRUCTURE were performed with the number of clusters (K) varying from 1 to 11, leading to 55 Structure outputs. We then estimated the number of subpopulations and the best output on the basis of the Evanno criterion [50].

Genetic dissimilarities between accessions were calculated using the simple matching coefficient in DARwin software [51]. Cluster analysis and dendrogram tree construction were performed based on dissimilarity matrices with the un-weighted pair-group method using arithmetic averages (UPGMA). Principal coordinate analysis was also used to reveal the relationships among the 250 accessions based on the above dissimilarity matrices, with the help of NTSYS-pc version2.1 software [43].

Basic statistics of genetic diversity including total number of alleles, and polymorphism information content (*PIC*) at each SSR locus according to the formula *PIC* = 1-∑*p_i_^2^* [52] where *p_i_* is the frequency of the *i*th allele, were carried out with PowerMarker v3.25 [53]. Genetic differentiation between landraces and modern varieties on a genome basis was detected with POPGENE software [54] using coefficients gene flow (*N_m_*), genetic distance (*GD*), genetic identity (*GI*), Shannon's information index (*I*) and coefficient of gene differentiation (*F_st_*). The genetic variation within and among populations of wheat accessions for different genomes was evaluated using analysis of molecular variance (AMOVA) implemented in Arlequin v3.11 software [55]. Due to the different sample sizes of the two sub-groups, an allele rarefaction method was used to standardize the allelic richness of samples [56].

Linkage disequilibrium (LD) between markers, including the pairwise estimated squared allele-frequency correlations (r^2^) and significance of each pair of loci [57], was calculated with the dedicated procedure of the TASSEL software [58]. In the process of LD estimation, SSR datasets were filtered for rare alleles with frequencies of less than 5% in the whole collection and computed using 100,000 permutations.

## Supporting Information

Figure S1 **Consensus genetic maps showing positions of the 512 SSR loci studied [source:** http://www.shigen.nig.ac.jp/wheat/komugi/top/top.jsp **].** Numbers on the left are genetic distances in centiMorgan.
(DOC)

Figure S2 **Comparative PIC distributions of SSR loci in the landrace and modern variety sub-groups for all 21 wheat chromosomes.** Blue curves show PIC trends in the modern variety sub-group, and red curves show trends for the landrace sub-group. Blue broken line means average PIC value of all SSR loci for the modern variety, and red broken line for the landrace. The detailed mean PIC values are listed at the bottom of each broken line. Genetic positions (cM) of SSR loci are from the Komugi wheat genetic resources database [http://www.shigen.nig.ac.jp/wheat/komugi/top/top.jsp].
(DOC)

Table S1 Detailed information of 250 accessions used in the study.
(XLS)

Table S2 List of 512 SSR loci used in the study.
(XLS)

Table S3 Chromosomal distribution of SSR loci used in the study.
(XLS)

Table S4 Molecular database of 512 SSR loci in 250 Chinese wheat genetic resources.
(XLS)
