# Supplementary material for: Genetic Diversity and Linkage Disequilibrium in Chinese Bread Wheat (Triticum aestivum L.) Revealed by SSR Markers
Source: PLoS One. 2011 Feb 18;6(2):e17279. doi: 10.1371/journal.pone.0017279 (PMC3041829; doi:10.1371/journal.pone.0017279)
Supplement: Figure S1 — Consensus genetic maps showing positions of the 512 SSR loci studied [source: http://www.shigen.nig.ac.jp/wheat/komugi/top/top.jsp ]. Numbers on the left are genetic distances in centiMorgan. (DOC) [file pone.0017279.s001.doc]

**Genetic Diversity and Linkage Disequilibrium in Chinese**

**Bread Wheat (*Triticum aestivum* L.) Revealed by SSR Markers**

**1A 1B 1D**

**2A 2B 2D**

**3A 3B 3D**

**4A 4B 4D**

**5A 5B 5D**

**6A 6B 6D**

**7A 7B 7D**

**Figure S1. Consensus genetic maps showing positions of the 512 SSR loci studied [source:** [**http://www.shigen.nig.ac.jp/wheat/komugi/top/top.jsp**](http://www.shigen.nig.ac.jp/wheat/komugi/top/top.jsp)**].** Numbers on the left are genetic distances in centiMorgan.
