# Supplementary material for: Genetic Diversity and Linkage Disequilibrium in Chinese Bread Wheat (Triticum aestivum L.) Revealed by SSR Markers
Source: PLoS One. 2011 Feb 18;6(2):e17279. doi: 10.1371/journal.pone.0017279 (PMC3041829; doi:10.1371/journal.pone.0017279)
Supplement: Figure S2 — Comparative PIC distributions of SSR loci in the landrace and modern variety sub-groups for all 21 wheat chromosomes. Blue curves show PIC trends in the modern variety sub-group, and red curves show trends for the landrace sub-group. Blue broken line means average PIC value of all SSR loci for the modern variety, and red broken line for the landrace. The detailed mean PIC values are listed at the bottom of each broken line. Genetic positions (cM) of SSR loci are from the Komugi wheat genetic resources database [http://www.shigen.nig.ac.jp/wheat/komugi/top/top.jsp]. (DOC) [file pone.0017279.s002.doc]

**Genetic Diversity and Linkage Disequilibrium in Chinese**

**Bread Wheat (*Triticum aestivum* L.) Revealed by SSR Markers**

**1A 1B**

**1D 2A**

**2B 2D**

**3A 3B**

**3D 4A**

**4B 4D**

**5A 5B**

**5D 6A**

**6B 6D**

**7A 7B**

**7D**

**Figure S2. Comparative PIC distributions of SSR loci in the landrace and modern variety sub-groups for all 21 wheat chromosomes.** Blue curves show PIC trends in the modern variety sub-group, and red curves show trends for the landrace sub-group. Blue broken line means average PIC value of all SSR loci for the modern variety, and red broken line for the landrace. The detailed mean PIC values are listed at the bottom of each broken line. Genetic positions (cM) of SSR loci are from the Komugi wheat genetic resources database [<http://www.shigen.nig.ac.jp/wheat/komugi/top/top.jsp>].
